# Costs, cost-effectiveness, and context

**DOI:** 10.1093/ajcn/nqac254

**Published:** 2022-10-03

**Authors:** Reina Engle-Stone, Katherine P Adams

**Affiliations:** Institute for Global Nutrition, University of California, Davis, Davis, CA, USA; Department of Nutrition, University of California, Davis, Davis, CA, USA; Institute for Global Nutrition, University of California, Davis, Davis, CA, USA; Department of Nutrition, University of California, Davis, Davis, CA, USA

See corresponding article on page 1303.

Nearly 5 decades after the International Nutritional Anemia Consultative Group published *Guidelines for the Eradication of Iron Deficiency Anemia* (1), identifying effective interventions and scalable delivery platforms to reduce iron deficiency and anemia among children remains a vital research question. In addition to evidence on impacts or benefits of intervention programs, program investment decisions require information on the resources (financial, human, and other) required to implement them; that these decisions should be informed by the relative costs and efficiency of alternative policy options for addressing anemia has also been recognized for some time (2). Compared with the literature on impact of iron interventions (including both beneficial effects and potential harms), however, there is sparse evidence on the costs and cost-effectiveness of iron deficiency and anemia control programs and how these relate to other nutrition and health investments.

Adding to the literature on cost-effectiveness of iron interventions among children, Akpan et al. (3) estimated cost-effectiveness of micronutrient powders (MNPs) and iron supplements for reducing disability-adjusted life years (DALYs) from anemia among children <2 y of age. Estimates of anemia reduction were drawn from a randomized trial of MNP or iron supplementation compared with placebo among children <2 y of age in Bangladesh (supplemented for 3 mo and followed for 9 mo); DALYs were calculated based on anemia cases averted. Health care costs incurred by participants were estimated from trial data, but intervention delivery costs were not available and so were drawn from unit costs (per child per year) for MNP delivery in Kenya and Rwanda. Calculated incremental cost-effectiveness ratios (ICERs) were $1645/DALY averted for iron supplements and $2400 for MNP, compared with placebo. The authors concluded that universal provision of MNP or iron supplements was not cost-effective compared with thresholds of $200 and $985, based on modeled health opportunity costs in Bangladesh and half the GDP per capita, respectively.

These results are useful for discussions around allocation of scarce resources available to fund health interventions. In the context of rural Bangladesh and if the policy objective is to most efficiently allocate resources to avert DALYs, then universal iron supplementation for children may not be a wise investment. However, several other factors may be useful to consider.

First, although the DALY is a convenient metric to estimate health burden and compare across conditions, a persistent critique is that DALYs fail to capture all potential dimensions of intervention benefits. In this example, disability weights for anemia do not capture all the potential effects of iron deficiency among children, or potential benefits of other micronutrients in MNPs. DALY weights exist for developmental intellectual disability; however, evidence is still inconsistent on the effects of iron and micronutrient interventions on child development (4). The authors did not model impacts on child cognition on the basis that no effect was observed on this outcome in the trial; including effects observed in other studies would improve cost-effectiveness, although it is not clear whether it would change the conclusion. A further consideration is that programs to address inadequate complementary feeding practices can reduce stunting and wasting, which are associated with increased mortality risk (5). Programs that both promote improved complementary feeding practices and distribute micronutrient supplements or MNPs were not the focus of this analysis; such programs may have additional benefits (and costs).

Evaluation of cost-effectiveness relative to a fixed threshold, as in this study, is useful for general categorization of interventions as cost-effective or not. However, given the lack of consensus on the most appropriate cost-effectiveness threshold and whether/how cost-effectiveness thresholds should be used for decision making, comparisons of cost-effectiveness with alternative intervention programs can also be instructive. Similar or greater costs per DALY averted ($1000–$5000) have been reported for interventions such as rural water supply/sanitation and cesarean delivery in low-income countries (6) and home gardens in Bangladesh (7). Other examples relevant to anemia prevention among children, both focused on MNP programs in Bangladesh, include $1557/DALY averted due to anemia (8) and $159/DALY averted due to anemia (considering mortality from severe anemia in addition to disability) (9). A priority for the research community should be to understand the factors driving differences in cost-effectiveness estimates and identify methodological best practices. Donors or governments can decide what they are willing to invest, given budget constraints, and what other priorities will drive investment decisions (e.g., equity may take precedence over cost-effectiveness).

A final consideration is whether targeting might be more cost-effective. This analysis focused on universal distribution of iron supplements or MNPs to all children within the specified age range (consistent with WHO guidelines). New modeling scenarios that examine both the impacts and costs of targeting the most vulnerable children would offer the opportunity to explore how the cost per DALY averted may differ for different program designs (e.g., targeting based on household or community characteristics compared with point-of-care anemia assessment).

The analysis also highlights some of the trade-offs faced by analysts in selecting inputs into cost-effectiveness analyses. For example, benefits and costs measured in research studies may be more accurate than estimates from program data, but they may not reflect programmatic reality in terms of costs or benefits. Similarly, is it preferable to use estimates from a single study conducted in the country of interest, or from a meta-analysis of studies in several countries? Critically, benefits and costs must “match.” That is, accuracy of cost-effectiveness estimates rests on costs reflecting the context in which impacts were generated, including aspects such as the frequency and duration of supplementation, mode of delivery, implementation scale, and context. In the article by Akpan et al., the lack of direct information on product distribution costs is a limitation. However, their extensive uncertainty modeling helps to overcome this limitation and guide interpretation of results. Given the uncertainty faced in most cost-effectiveness studies, conducting detailed sensitivity analyses and uncertainty modeling, as was done in this analysis, is a best practice that should be encouraged.

Collecting high-quality cost data should also be prioritized. Specifically, researchers should endeavor to collect activity-based cost data including the unit cost and quantity of all inputs required to carry out each activity associated with an intervention, from start-up through implementation and follow-up (10). Although in the context of a trial it can be challenging to disentangle research costs from intervention costs, generating high-quality cost estimates is possible with a well-planned costing strategy in which resource use and costs are continuously collected via expenditure records, monitoring data, interviews, etc. and categorized as programmatic, research, or shared. Improved cost data, derived from both trial and programmatic contexts, will encourage and inform cost-effectiveness analyses and help governments and donors make more informed decisions.

Cost-effectiveness analyses are useful to guide program investments, but are still relatively scarce in the nutrition literature and are often limited by data availability, particularly on costs. Incorporating best practices for cost data collection into nutrition research may increase the availability and quality of cost-effectiveness analyses; collection of quality data on intervention impacts and costs from programmatic settings is similarly, if not more, important, to ensure that estimates reflect program conditions. Developing a portfolio of public health interventions that fits within a country's budget constraints, reflects policy priorities, and provides good value for money is complex and context-specific. High-quality cost-effectiveness analyses, with targeted sensitivity analyses to address data gaps, can help guide the development of such portfolios. Ultimately, policy makers must decide how investments in child anemia compare with other investment priorities, and how much they are willing and able to invest.

## Notes

The authors reported no funding received for this study.
